# Assessment of cricket frass as a sustainable organic fertilizer: Effects on seedling establishment, growth and fruit yield of zucchini (*Cucurbita pepo*)

**DOI:** 10.1371/journal.pone.0351645

**Published:** 2026-07-06

**Authors:** Clarcky Andrianorosoa Ony, Cédrique Lova Solofondranohatra, Tanjona Ramiadantsoa, Andrianjaka Ravelomanana, Tantely Maminiaina Razafimbelo, Andry Andriamananjara, Sakib Burza, Sabrina Simon, Mahardika Putra Purba, Brian L. Fisher

**Affiliations:** 1 Madagascar Biodiversity Center, Antananarivo, Madagascar; 2 Agriculture-Elevage-Environnement, Université d’Antananarivo, Madagascar; 3 Laboratoire des RadioIsotopes, Antananarivo, Madagascar; 4 Health In Harmony, Farafangana, Madagascar; 5 London School of Hygiene and Tropical Medicine, London, United Kingdom; 6 Department of Forestry, State Agricultural Polytechnic of Kupang, Indonesia; 7 Department of Entomology, California Academy of Sciences, San Francisco, California, United States of America; Arizona State University, UNITED STATES OF AMERICA

## Abstract

Inherently poor soil quality, compounded by widespread soil degradation from unsustainable farming practices, continues to constrain agricultural productivity in Madagascar. As a result, fertilizer application has become essential for improving crop yields. This study assessed the effects of cricket frass fertilizer (CFF) from *Gryllus* madagascariensis on zucchini (*Cucurbita pepo*) production, in comparison with cattle manure (CM) and NPK, across two contrasting sites in southeastern Madagascar: Tsaratanàna, a relatively nutrient-rich coastal site, and Namohora, a nutrient-poor inland site. Seven fertilizer treatments were evaluated: an unfertilized control, CM (3.75 g N/plant), NPK (3.75 g N/plant), CFF at the baseline dose (3.75 g N/plant), ¼ × CFF (0.94 g N/plant), 2 × CFF (7.50 g N/plant), and a CM + CFF combination (7.50 g N/plant total). Plant survival was highest with ¼ × CFF in Tsaratanàna and with 2 × CFF in Namohora. Plant height and leaf number generally increased with increasing CFF dose at both sites. Yield responses were site-dependent: NPK produced the highest yield in Tsaratanàna, whereas 2 × CFF produced the highest yield in Namohora. Overall, CFF improved zucchini survival, growth, and yield relative to the unfertilized control. CFF performance relative to cattle manure varied across sites but generally remained comparable or superior, whereas its performance relative to NPK was not consistently superior across sites. These findings indicate that CFF is a promising organic fertilizer for zucchini, particularly under nutrient-poor conditions.

## 1. Introduction

Agriculture in Madagascar faces significant constraints due to both inherently poor soil quality and ongoing soil degradation. The island’s dominant soil type, ferralitic soil, is typically acidic and deficient in essential nutrients and organic matter, while also containing high levels of iron and aluminum that limit nutrient availability and root development [1]. These natural limitations are exacerbated by unsustainable agricultural practices, deforestation, and erosion, all of which contribute to declining soil fertility [2]. As approximately 75% of the Malagasy population depends on agriculture for their livelihoods, this degradation directly threatens food security and rural incomes [3].

To mitigate soil fertility loss and enhance productivity, farmers in Madagascar rely on both organic and chemical fertilizers. Organic fertilizers, such as animal manure and plant-based composts, are widely used due to their accessibility and benefits for soil health, including improved structure, microbial activity, and water retention [4,5]. Cattle manure (CM) is the most commonly used organic amendment, yet its nutrient content is relatively low due to nutrient losses during animal metabolism and manure handling [6,7]. On the other hand, chemical fertilizers such as NPK are highly effective due to their nutrient concentration, but they are costly, environmentally damaging, and largely inaccessible to smallholder farmers [8,9].

In this context, insect frass, a mixture of feces, uneaten feed, and molted exoskeletons, has emerged as a novel organic fertilizer, particularly in regions where insect farming is gaining traction. Frass is rich in nitrogen and bioactive compounds such as chitin, and it has shown promise for improving plant performance and soil microbial activity [10,11]. A growing number of studies have documented the agronomic benefits of black soldier fly frass (BSFF), which has been shown to enhance plant growth and yield in maize, soybean, ryegrass, spinach, sweet potato, and even to support forest restoration as well [12–19].

More recently, interest has turned toward cricket frass, which offers similarly promising effects on crops and soil but remains comparatively less-studied. Studies on cricket frass from *Acheta domesticus*, *Gryllus bimaculatus*, and *Gryllus madagascarensis* have reported significant improvements in spring onion growth and yield [20], biomass in Chinese kale and spider plant [21,22], and biostimulant effects on tomato seedlings [23]. In Madagascar, field trials using frass from *G. madagascarensis* showed that high application rates improved the survival, growth, and yield of green beans (*Phaseolus vulgaris*) under nutrient-poor conditions [24].

Recently, insect farming has expanded in Madagascar, generating insect frass. Insect frass is rich in nitrogen and other nutrients and shows promise as a sustainable organic fertilizer [11]. In particular, cricket frass fertilizer (CFF) produced from *G. madagascarensis*—a species now farmed for food and feed—may offer a viable alternative to conventional fertilizers. Preliminary research in Madagascar showed that CFF application significantly improved survival, growth, and yield in green beans (*Phaseolus vulgaris*) [24]. Despite growing global interest in insect-derived fertilizers, very little research has focused on CFF, particularly under Malagasy field conditions.

Zucchini, with its short cycle and nutritional benefits, can play a small but meaningful role in rural livelihoods and diets. One reason zucchini (and related squashes) appeal to Malagasy farmers is their multi-purpose use. Both the young fruits and the tender leaves are consumed. This dual use (fruits and leaves) makes zucchini valuable for home consumption, especially in combating nutritional deficiencies. Even zucchini flowers are sometimes picked and boiled or added to dishes [25–27]. Vegetables such as zucchini are typically cultivated in home gardens or small plots, primarily to supplement household diets and sometimes generate additional income [25,26]. Organized initiatives have demonstrated that enhanced market gardening—including zucchini production—can significantly boost off-season earnings, potentially tripling household income through the sale of fresh produce [28].

Although insect-derived fertilizers such as cricket frass fertilizer (CFF) have recently attracted attention for their potential agronomic and environmental benefits, research in Madagascar remains limited and largely restricted to green beans. In contrast, little to no work has examined the effects of CFF on zucchini (*Cucurbita pepo*), a crop of nutritional and economic importance whose dual-purpose use—both fruits and tender leaves—enhances household food security and income generation. This represents a critical gap, as zucchini’s short growth cycle and multi-purpose value make it an ideal candidate for testing innovative fertilization strategies. Furthermore, while traditional organic amendments such as cattle manure are widely used, their variable nutrient content and slow nutrient release may constrain productivity. By comparison, CFF, which is richer in readily available nitrogen and other essential nutrients, may provide a comparative advantage, offering both improved crop performance and a more sustainable alternative under smallholder farming conditions. Yet, no study has tested its performance on zucchini under different soil fertility conditions, a gap that is critical for understanding its potential across the diverse farming systems of Madagascar.

Here we conducted a field experiment to evaluate the effects of cricket frass fertilizer (CFF) on the survival, growth, and yield of zucchini (*Cucurbita pepo var. cylindrica*). Given its agronomic and economic importance, zucchini serves as an ideal model crop to assess fertilizer efficacy under open-field conditions. The experiment was carried out at two contrasting sites in southeastern Madagascar—Tsaratanàna (coastal) and Namohora (inland)—that differ significantly in soil fertility. We compared the effects of multiple doses of CFF to those of cattle manure and NPK, the two most commonly used fertilizers in the region. Our findings aim to provide evidence-based guidelines for farmers planting zucchini and other cucurbits in Madagascar while contributing to the broader understanding of insect frass as a sustainable soil amendment in low-input agricultural systems.

## 2. Materials and methods

### 2.1. Site description

A field experiment was carried out in Farafangana, located in the southeastern part of Madagascar, under open-field conditions from May to August 2024. The experiment was performed in a farm field managed by Health In Harmony (HIH), a Non-Governmental Organization working on a conservation program in Farafangana. Two contrasting sites were used: Tsaratanàna, a coastal and relatively nutrient-rich site (23° 02′ 20′′ S, 47° 45′ 53′′ E), and Namohora, an inland and nutrient-poor site (23° 04′ 37′′ S, 47° 43′ 10′′ E); their key agroecological attributes are presented in Table 1. During the experimental period, mean daily temperature recorded by dataloggers ranged from 18 to 27 °C at both sites, and mean annual rainfall in the region is approximately 2,500 mm [29]. Baseline soil analyses confirmed the contrast between the sites: Tsaratanàna had higher organic matter, cation exchange capacity, and available phosphorus, whereas Namohora had lower fertility overall (Table 2). All necessary permits for the described fieldwork were obtained from the Ministry of Environment and Sustainable Development of Madagascar.

**Table 1:** Comparative summary of key agroecological attributes of the two study sites.

Attributes	Tsaratanàna	Namohora
**COORDINATES**		
Geographic coordinates	23°02′20″S, 47°45′53″E	23°04′37″S, 47°43′10″E
Altitude (m)	2.3	9.2
Location	Coastal	Inland
Administrative location	Farafangana, southeastern Madagascar	Farafangana, southeastern Madagascar
**CLIMATE**		
Mean daily temperature range (°C)	18–27	18–27
Mean annual rainfall (mm)	~2 500	~2 500
Experimental period	May – August 2024	May – August 2024
**SOIL CHARACTERISTICS**		
Baseline land use	Fallow	Fallow
Soil texture	Clay soil	Clay soil
Overall fertility	Higher (nutrient-rich)	Lower (nutrient-poor)
Soil organic matter	Higher	Lower
Cation Exchange Capacity (CEC)	Higher	Lower
Soil pH	Highly acidic	Moderately acidic

Soil characteristics are based on baseline soil analyses. Temperatures are recorded by dataloggers. Rainfall data are from regional sources [29].

**Table 2:** Soil physico-chemical properties before fertilizer application in Tsaratanàna and Namohora.

	Tsaratanàna		Namohora	
**Parameters**	**Value**	**Level**	**Value**	**Level**
pH (H2O)	5.38	Highly acidic	5.71	Moderately acidic
Organic matter (%)	7.03	Very rich	2.84	Medium
C/N	16.6	Satisfying	15.7	Satisfying
CEC (cmol(+)/kg)	16.7	Medium	6.6	Low
Nitrogen (%)	0.25	Very poor	0.11	Very poor
Phosphorus (mg/kg)	23.69	Rich	4.18	Poor
Potassium (mg/kg)	31	Very poor	24	Very poor
Calcium (mg/kg)	105	Very poor	56	Very poor
Magnesium (mg/kg)	102	Poor	44	Poor
Sodium (mg/kg)	10	–	7	–
Clay (%)	62	–	56	–
Silt (%)	23	–	17	–
Sand (%)	15	–	27	–

C/N: carbon to nitrogen ratio; CEC: Cation Exchange Capacity

### 2.2. Treatments

The experiment included three fertilizers: cricket frass fertilizer (CFF), cattle manure (CM), and NPK 11-22-16. We obtained CFF from *G. madagascarensis* rearing. *G. madagascarensis* is farmed in a plastic box at EXA Farm in Antananarivo, where crickets are fed with chicken feed. They are harvested after 28 days, and cricket frass fertilizer (CFF) is collected. CFF consists of a mixture of dead crickets, leftover feed, and cricket feces. The CM and NPK came from a local store in Antananarivo. The NPK content (percentage of nitrogen, phosphorus, and potassium) of the fertilizers is presented in Table 3. We based the baseline fertilizer dose on HIH recommendation, using the nitrogen content of cattle manure typically applied to zucchini (131 kg N/ha) in the Region, which is close to the recommended (120 kg N/ha) [30]. In accordance with HIH recommendation, cattle manure (CM) should be applied at a dose of 375 g per plant (250 g of CM during sowing, and 125 g of CM one month after the sowing), which contains 3.75 g of nitrogen. Thus, we applied the same amount of nitrogen per plant (3.75 g) using cattle manure, NPK, and CFF. The amount of 3.75 g of nitrogen is equivalent to 375 g of CM, 34.13 g of NPK, and 93.75 g of CFF (Table 3). As some studies highlighted the toxic effect of frass on plants [31,32], we also assessed two additional doses of CFF: 1/4 of the baseline and double the baseline, in addition to comparing the three fertilizers at the baseline dose. Furthermore, we included a treatment combining one baseline dose of CFF with one baseline dose of cattle manure to assess any possible interaction between the two fertilizers. Finally, the control treatment consisted of unfertilized soil. In sum, the seven treatments were: control (no fertilizer), cattle manure baseline, NPK baseline, CFF, ¼ × CFF, CFF double the baseline, and a mixture of cattle manure baseline and CFF. We applied dolomite with each treatment. Dolomite was uniformly applied to all experimental plots at a dose of 25 g/m^2^, regardless of the type of fertilizer treatment, in order to ensure baseline homogeneity across the trial. This approach allowed fertilizer type and application dose to remain the only variables distinguishing the plots, thereby facilitating reliable comparisons among treatments. Because treatments were standardized on an N-equivalent basis, the amounts of elemental P, K, and selected secondary nutrients differed substantially among fertilizers and doses. To make these differences explicit, Table 3 presents the chemical composition of the fertilizers and Table 4 presents the calculated nutrient inputs per plant for each treatment. Accordingly, treatment effects should be interpreted as the performance of the fertilizer materials as applied, rather than as responses to fully nutrient-balanced treatments.

**Table 3:** Chemical composition of the three fertilizers used in the experiment.

Fertilizers	N total (%)	P (%)	K (%)	Ca (%)	Mg (%)	Na (%)
CFF	4.0	0.3	2.2	2.5	0.9	0.4
CM	1.0	0.3	2.5	0.2	0.1	0.1
NPK	11.0	9.6	13.3	0	0	0

Fertilizers: cricket frass fertilizer (CFF), cattle manure (CM), NPK

**Table 4:** Quantity of fertilizer added in grams for each treatment.

Treatments	N(g/plant)	P(g/plant)	K(g/plant)	Ca(g/plant)	Mg(g/plant)	Na(g/plant)	Fertilizer applied(g/plant)
Control	0.00	0.00	0.00	0.00	0.00	0.00	0.00
CM	3.75	1.14	9.34	0.75	0.38	0.38	375.00
NPK	3.75	3.27	4.53	0.00	0.00	0.00	34.13
CFF	3.75	0.28	2.06	2.34	0.84	0.38	93.75
¼ × CFF	0.94	0.07	0.52	0.59	0.21	0.09	23.44
2 × CFF	7.50	0.56	4.13	4.69	1.69	0.75	187.50
CM + CFF	7.50	1.42	11.4	3.09	1.22	0.76	468.75

Treatments: control, cattle manure (CM), NPK, cricket frass fertilizer (CFF), ¼ × CFF, 2 × CFF, and CFF + CM.

### 2.3. Soil preparation and experimental design

Due to the limited availability of references on zucchini cultivation in the Region, we followed the HIH protocol, which has guided local production for the past seven years. The protocol involved weed removal, soil tillage to 20 cm, and the preparation of 30 cm-high beds with 20 cm-diameter holes spaced 60 cm apart. Fertilizer and dolomite were incorporated into each hole, and two days later, two zucchini seeds were sown per hole.

**Fig 1:**
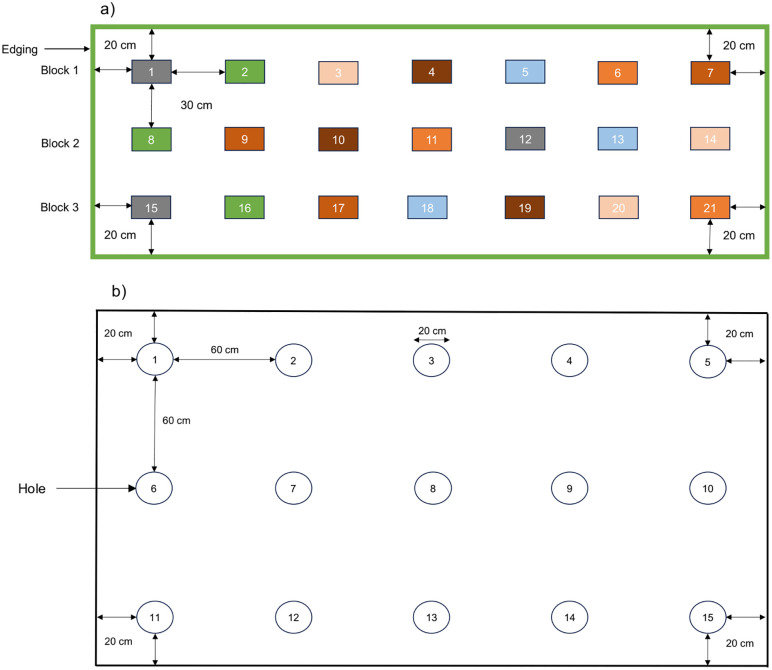
Experimental design and within-plot layout. **(a)** Experimental layout following a randomized complete block design (RCBD) with 3 blocks and 7 plots per block, where each color represents a treatment. The green rectangle indicates a border row of zucchini plants used to minimize edge effects. **(b)** Within-plot arrangement, showing a spacing of 60 cm between planting holes and 20 cm between each hole and the plot edge. Each plot contained 15 planting holes.

The experiment was laid out in a randomized complete block design (RCBD) with three replications and seven treatments (Fig 1). Thus, one block consisted of seven plots relative to the seven treatments. Each plot (3.8 m × 2.2 m) contained 15 holes, for a total of 1,260 plants (30 plants × 3 replicates × 7 treatments × 2 sites), and the distance between plot was 30 cm. While a higher number of replicates would have strengthened statistical power, three replicates within an RCBD framework are commonly adopted in field fertilization studies, as the blocking structure helps account for spatial heterogeneity and yields a reliable estimate of experimental error [33]. We tagged each individual to facilitate data collection. A row of zucchini crops was planted around the borders of the field, 20 cm away from the plots, to prevent edge effects. Irrigation was applied twice daily (15 l/m^2^), and Pyripro (0.1%) was sprayed at 0.08 l/m^2^ every two weeks for pest control. To assess experimental variability across replicates, coefficients of variation (CV) were calculated for the main response variables. CVs for plant height, leaf number, fruit length, and fruit diameter ranged from 14 to 46%, indicating moderate experimental variability considered acceptable for field trials of this type [33] (S1 Table). Higher CVs were generally associated with low-input treatments, where reduced fertilization likely increased sensitivity to micro-environmental heterogeneity. CVs for fruit yield were higher (44–101%), reflecting the inherent variability of yield components under field conditions [34,35] (S1 Table). Plant survival was not included in the CV assessment, as this variable is binary in nature and its variability reflects individual plant outcomes rather than spatial heterogeneity across plots. Despite this variability, treatment rankings remained consistent across replicates and sites, supporting the reliability of the observed treatment effects.

### 2.4. Data collection

Soil samples were collected at the start of the experiment and after harvest. During the setup, samples were taken from a 20 cm depth at five points across the experimental field (four corners and the center). This depth was chosen as it represents the main root zone of zucchini and the layer most influenced by fertilizer application and organic matter dynamics. Approximately 250 g of soil from each point were composited for homogeneity, and 250 g of the mixture were used for analysis. At harvest, soil samples were collected from three plots per treatment, using the same five-point sampling pattern. Samples from each plot were composited, and 250 g of each composite were analyzed in the laboratory. Soil samples were dried at 40 °C and sieved to 2 mm. Soil pH was measured in water (soil:solution = 1:2.5) using a glass electrode pH meter. Organic carbon and organic matter were determined by the Walkley–Black method, and total nitrogen by the Kjeldahl method. Available phosphorus was analyzed using the Bray II procedure. Exchangeable K, Ca, Mg, and Na were extracted with 1 N ammonium acetate, and total cation exchange capacity (CEC) was determined after base extraction, ethanol leaching, and KCl rinsing. Agronomic parameters, including plant survival, plant height, and leaf number, were recorded biweekly from two to seven weeks after sowing. Measurements continued throughout the study period until either the first harvest or plant mortality occurred. Seedling survival (%) was calculated for each plot as the number of live plants present at the start of harvest divided by the number of seeds sown in that plot, multiplied by 100. We measured the plant total height from ground level to the tallest point using a ruler, and the final height values were used for statistical analysis. At harvest time, we collected data on fruit diameter and fruit length using a measuring tape. We weighed the fruit using a scale (Dahongying ACS-A9) which has a precision of 5 grams.

### 2.5. Data analysis

Prior to analysis, data normality was assessed using the Shapiro–Wilk test and homogeneity of variances using Levene’s test. Survival percentages were compared among treatments using proportion tests followed by pairwise proportion tests with Bonferroni-adjusted p-values. Because plant height, leaf number, fruit diameter, fruit length, and yield did not meet parametric assumptions, these variables were analyzed using Kruskal–Wallis tests followed by Dunn’s multiple-comparison tests with Bonferroni correction. To assess whether fertilizer responses differed between sites, we fitted generalized linear models (GLMs) with site, treatment, and their interaction as fixed effects. Models were fitted using distribution families and link functions appropriate for each response variable: a Gamma distribution with a log link for continuous traits (height, diameter, length, and yield), a Poisson distribution with a log link for leaf number, and a binomial distribution with a logit link for survival. Significant terms were further analyzed using post-hoc pairwise comparisons of estimated marginal mean via the emmeans package. All analyses were performed in R version 4.5.2 [36], with statistical significance defined as p < 0.05.

## 3. Results

### 3.1. Soil analysis

Post-harvest soil properties varied among treatments at both sites (Tables 5–8). Because these soil variables were obtained from composite samples, they are presented descriptively and were not subjected to formal statistical comparison among treatments. At Tsaratanàna, soil pH spanned a range of 2.20 units across treatments, with the widest contrasts observed between NPK and CFF + CM. Available phosphorus and potassium showed the largest absolute differences among treatments, reaching up to 115.22 mg/kg and 191 mg/kg respectively, while nitrogen varied more modestly across treatments (Tables 5–8). At Namohora, soil pH ranged across 1.85 units, with the control recording the highest value and CFF treatment the lowest. Available phosphorus and potassium again showed the greatest treatment-related variation, peaking at 121.22 mg/kg and 1290 mg/kg respectively (Tables 5–8).

**Table 5:** Soil physico-chemical properties after zucchini harvesting in Tsaratanàna.

Treatments	pH (H2O)	Organic matter (%)	CEC (cmol(+)/kg)	Clay (%)	Silt (%)	Sand (%)
Control	6.97	3.42	13.3	44	42	14
CM	6.43	2.64	13.3	46	34	20
NPK	5.73	3.18	14.1	44	40	16
¼ × CFF	6.72	3.82	12.1	50	38	12
CFF	5.91	3.82	13.6	46	40	14
2 × CFF	5.91	4.56	12.6	40	38	22
CFF + CM	7.93	5.27	14.2	52	32	16

Soil pH, organic matter, cation exchange capacity, percentages of clay, silt, and sand after application of different fertilizers: control, cattle manure (CM), NPK, cricket frass fertilizer (CFF), ¼ × CFF, 2 × CFF, and CFF + CM.

**Table 6:** Soil nutrient contents after zucchini harvesting in Tsaratanàna.

Treatments	N (%)	P (mg/kg)	K (mg/kg)	Ca (mg/kg)	Mg (mg/kg)	Na (mg/kg)
Control	0.21	1.40	68	551	334	14
CM	0.26	11.81	137	602	395	58
NPK	0.21	115.22	138	201	222	93
¼ × CFF	0.23	6.33	101	682	586	36
CFF	0.26	28.51	141	461	324	70
2 × CFF	0.29	46.51	191	511	355	87
CFF + CM	0.32	18.45	147	582	385	66

Soil nitrogen content (N), phosphorus (P), potassium (K), calcium (Ca), magnesium (Mg), sodium (Na) after application of different fertilizers: control, cattle manure (CM), NPK, cricket frass fertilizer (CFF), ¼ × CFF, 2 × CFF, and CFF + CM.

**Table 7:** Soil physico-chemical properties after zucchini harvesting in Namohora.

Treatments	pH (H2O)	Organic matter (%)	CEC (cmol(+)/kg)	Clay (%)	Silt (%)	Sand (%)
Control	7.37	1.39	5.8	42	32	26
CM	6.61	2.49	6.1	38	32	30
NPK	5.59	1.64	8.9	38	32	30
¼ × CFF	5.94	1.69	14.5	42	30	28
CFF	5.78	2.45	10.8	36	30	34
2 × CFF	5.52	1.95	8.3	32	32	35
CFF + CM	5.52	3.36	11.5	40	26	34

Soil pH, organic matter, cation exchange capacity, percentages of clay, silt, and sand after application of different fertilizers: control, cattle manure (CM), NPK, cricket frass fertilizer (CFF), ¼ × CFF, 2 × CFF, and CFF + CM.

**Table 8:** Soil nutrient contents after zucchini harvesting in Namohora.

Treatments	N (%)	P (mg/kg)	K (mg/kg)	Ca (mg/kg)	Mg (mg/kg)	Na (mg/kg)
Control	0.11	3.15	1036	716	20	25
CM	0.18	6.22	724	385	31	72
NPK	0.15	121.22	822	485	32	262
¼ × CFF	0.12	5.01	942	351	24	39
CFF	0.22	48.13	1017	485	58	71
2 × CFF	0.15	35.11	1290	551	41	80
CFF + CM	0.20	36.22	1231	567	48	77

Soil nitrogen content (N), phosphorus (P), potassium (K), calcium (Ca), magnesium (Mg), sodium (Na) after application of different fertilizers: control, cattle manure (CM), NPK, cricket frass fertilizer (CFF), ¼ × CFF, 2 × CFF, and CFF + CM.

### 3.2. Seedling survival percentage

Seedling survival percentage responded clearly to fertilization at both sites (Table 9). At Tsaratanàna, unfertilized seedlings survived poorly (22.22%), while fertilized treatments achieved survival rates between 58.89% (CFF) and 75.56% (¼ × CFF), between 37 and 53 percentage points higher than the control. Among fertilized treatments, only ¼ × CFF and CFF differed significantly from each other, with the remaining treatments falling in between. At Namohora, no seedling survived in the control, while fertilized treatments ranged from 51.11% (¼ × CFF) to 91.11% (2 × CFF), a span of 40 percentage points, with ¼ × CFF and CM recording significantly lower survival than the remaining fertilized treatments. A significant site × fertilizer interaction (GLM, p < 0.05) indicated that treatment responses differed between sites. No site differences were detected for most treatments, except for ¼ × CFF and 2 × CFF, which showed contrasting responses depending on location.

**Table 9:** Survival percentage of zucchini seedlings.

Sites	Treatments	Means	SE
Tsaratanàna	Control	22.22^d^	4.41
Tsaratanàna	¼ × CFF	75.56^a^	4.56
Tsaratanàna	CM	62.22^ab^	5.14
Tsaratanàna	NPK	65.56^ab^	5.04
Tsaratanàna	CFF	58.89^bc^	5.22
Tsaratanàna	2 × CFF	65.56^ab^	5.04
Tsaratanàna	CFF + CM	64.44^ab^	5.07
Namohora	Control	0.00^d^	0
Namohora	¼ × CFF	51.11^c^	5.30
Namohora	CM	53.33^c^	5.29
Namohora	NPK	68.89^b^	4.91
Namohora	CFF	72.22^b^	4.75
Namohora	2 × CFF	91.11^a^	3.02
Namohora	CFF + CM	76.67^b^	4.48

Within each site, values followed by different letters differ significantly according to pairwise proportion tests with Bonferroni correction (p < 0.05). SE: standard error. Treatments: control, cattle manure (CM), NPK, cricket frass fertilizer (CFF), ¼ × CFF, 2 × CFF, and CFF + CM.

### 3.3. Seedling growth

**Fig 2:**
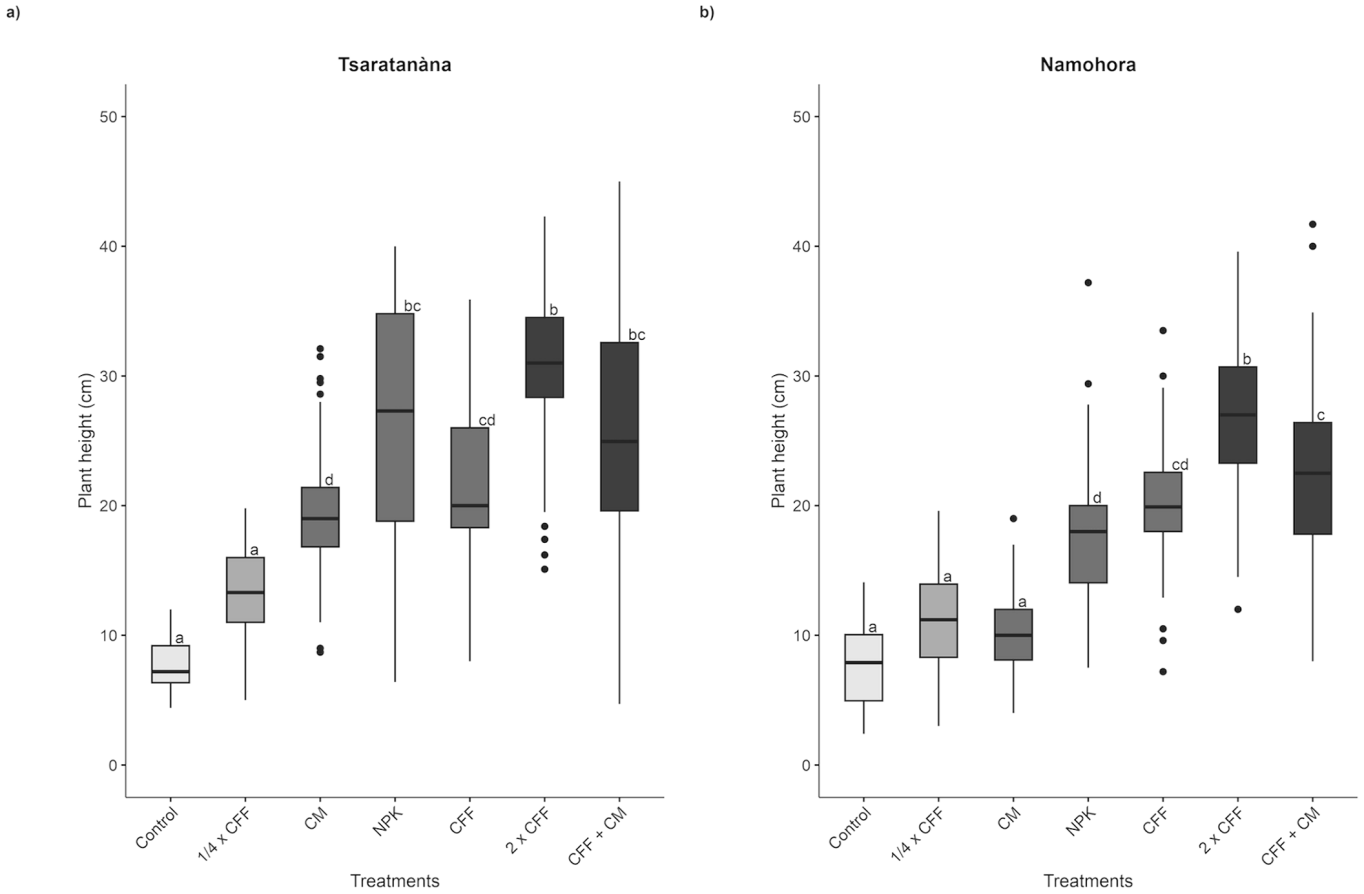
Plant height of zucchini under different fertilizer treatments. **(a)** Coastal site (Tsaratanàna); **(b)** Inland site (Namohora). Treatments: control, cattle manure (CM), NPK, cricket frass fertilizer (CFF), ¼ × CFF, 2 × CFF, and CFF + CM. Different letters indicate significant differences according to Dunn’s multiple-comparison tests with Bonferroni correction (p < 0.05). Colors represent treatments with similar nitrogen content.

Plant height was significantly affected by fertilizer treatment (Kruskal–Wallis test, p < 0.05) (Fig 2). At Tsaratanàna, the highest fertilized treatment exceeded the control by 22.68 cm, with ¼ × CFF and CM recording significantly lower heights than the remaining fertilized treatments. At Namohora, the corresponding difference was 19.34 cm, with a similar pattern of treatment differentiation (Fig 2). The effect of site on plant height varied among fertilizer treatments, as indicated by a significant site × fertilizer interaction (GLM, p < 0.001). No significant differences between sites were observed for the control, CM, and CFF (p > 0.05). In contrast, ¼ × CFF, 2 × CFF, NPK, and CFF + CM showed significant site effects (p < 0.05 to p < 0.001), with consistently higher plant height in Tsaratanàna than in Namohora.

**Fig 3:**
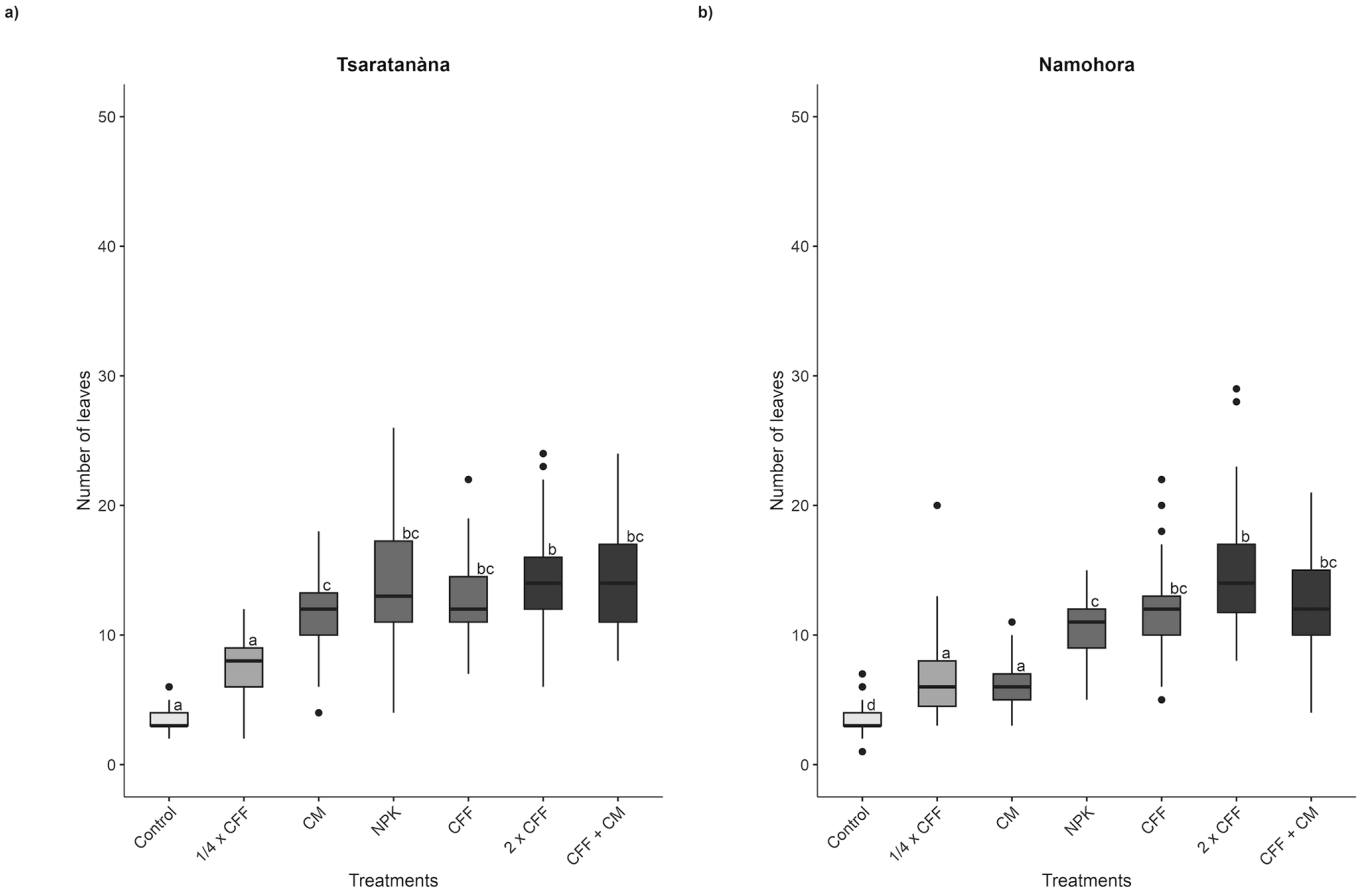
Leaf number of zucchini under different fertilizer treatments. **(a)** Coastal site (Tsaratanàna); **(b)** Inland site (Namohora). Treatments: control, cattle manure (CM), NPK, cricket frass fertilizer (CFF), ¼ × CFF, 2 × CFF, and CFF + CM. Different letters indicate significant differences according to Dunn’s multiple-comparison tests with Bonferroni correction (p < 0.05). Colors represent treatments with similar nitrogen content.

The number of leaves per plant was significantly affected by fertilizer treatment (Kruskal–Wallis test, p < 0.05) (Fig 3). At both sites, the highest fertilized treatments exceeded the control by 11 leaves, with CM at Tsaratanàna and ¼ × CFF and CM at Namohora recording significantly lower values than the best-performing treatments (Fig 3). The effect of site on the number of leaves differed across fertilizer treatments (GLM, p < 0.001). No significant site differences were observed for the control, CM, and CFF (p > 0.05). In contrast, ¼ × CFF, 2 × CFF, NPK, and CFF + CM showed significant site effects (p < 0.05 to p < 0.001), with higher leaf numbers in Tsaratanàna than in Namohora.

### 3.4. Zucchini yield

Fruit yield was significantly affected by fertilizer treatment (Kruskal–Wallis test, p < 0.05; Table 10), with no yield recorded in the control at either site. At Tsaratanàna, the range among fertilized treatments spanned 11.58 t/ha, while at Namohora it reached 11.55 t/ha, with CM and ¼ × CFF consistently underperforming relative to the other fertilized treatments at both sites (Table 10). The effect of site on yield varied among fertilizer treatments, as indicated by a significant site × fertilizer interaction (GLM, p < 0.001). No significant differences between sites were observed for ¼ × CFF, 2 × CFF, and CFF + CM (p > 0.05). In contrast, CM, CFF, and NPK showed significant site effects (p < 0.05 to p < 0.001). Yield was higher in Tsaratanàna for CM and NPK, while CFF showed slightly higher yield in Namohora.

**Table 10:** Fruit diameter, length, and yield of zucchini.

Sites	Treatments	Fruit diameter (cm)		Fruit length (cm)		Yield (t/ha)	
		Means	SE	Means	SE	Means	SE
Tsaratanàna	Control	0^e^	0	0^e^	0	0.00^e^	0.00
Tsaratanàna	¼ × CFF	3.95^a^	0.13	9.00^a^	0.28	0.89^c^	0.14
Tsaratanàna	CM	4.84^abd^	0.19	11.58^c^	0.42	3.24^b^	0.64
Tsaratanàna	NPK	5.95^c^	0.20	16.12^b^	0.46	12.47^a^	0.47
Tsaratanàna	CFF	4.73^bcd^	0.15	12.06^b^	0.37	3.95^b^	0.82
Tsaratanàna	2 × CFF	5.77^bc^	0.21	14.64^b^	0.48	10.67^a^	0.74
Tsaratanàna	CFF + CM	5.72^ad^	0.22	14.88^c^	0.57	11.53^a^	1.66
Namohora	Control	0.00^e^	0.00	0.00^e^	0.00	0.00^d^	0.00
Namohora	¼ × CFF	4.89^ab^	0.78	9.18^ab^	0.40	0.56^c^	0.24
Namohora	CM	3.72^b^	0.13	8.19^b^	0.36	0.31^c^	0.12
Namohora	NPK	4.36^ab^	0.12	12.08 cd	0.30	6.49^ab^	0.70
Namohora	CFF	4.48^ac^	0.10	11.07 cd	0.25	4.06^b^	0.47
Namohora	2 × CFF	5.17^c^	0.11	13.40^c^	0.34	11.86^a^	0.86
Namohora	CFF + CM	4.76^ab^	0.13	12.53^ad^	0.35	7.46^ab^	0.58

Within each site, values followed by different letters are significantly different based on Dunn’s test with Bonferroni correction (p < 0.05). SE: standard error. Treatments: control, cattle manure (CM), NPK, cricket frass fertilizer (CFF), ¼ × CFF, 2 × CFF, and CFF + CM.

Fertilization significantly affected fruit diameter at both sites (Kruskal–Wallis test, p < 0.05; Table 10). In both Tsaratanàna and Namohora, no fruits were obtained in the control treatment, resulting in no measurable fruit diameter. Among fertilized treatments, the range spanned 2.00 cm at Tsaratanàna and 1.45 cm at Namohora, with ¼ × CFF and CM recording the lowest values at their respective sites (Table 10). The effect of site on fruit diameter varied among fertilizer treatments, as indicated by a significant site × fertilizer interaction (GLM, p < 0.001). No significant site differences were observed for CM, ¼ × CFF, CFF, and CFF + CM (p > 0.05). In contrast, NPK and 2 × CFF showed significant site effects (p < 0.05), with larger fruit diameter in Tsaratanàna than in Namohora.

Fruit length was significantly affected by fertilizer treatment (Kruskal–Wallis test, p < 0.05) (Table 10). In both sites, Tsaratanàna and Namohora, no fruits were obtained in the control treatment, resulting in no measurable fruit length. Among fertilized treatments, the range spanned 15.23 cm at Tsaratanàna and 5.21 cm at Namohora, with ¼ × CFF and CM recording the lowest values at their respective sites (Table 10). The effect of site on fruit length varied among fertilizer treatments, as indicated by a significant site × fertilizer interaction (GLM, p < 0.001). No significant differences between sites were observed for CM and CFF (p > 0.05). In contrast, ¼ × CFF, 2 × CFF, NPK, and CFF + CM showed significant site effects (p < 0.05 to p < 0.001), with higher fruit length in Tsaratanàna than in Namohora.

## 4. Discussion

This study expands upon our previous work on cricket frass fertilizer (CFF) in green beans [24], by testing its performance on a different crop (zucchini) and under two distinct agroecological conditions. We introduced nitrogen-equivalent dosing across seven fertilizer treatments, including combinations of CFF and cattle manure, and assessed comparative impacts on plant survival, growth, and yield. Our results suggest that CFF improves the survival, growth, and yield of zucchini grown across two contrasting sites, Tsaratanàna and Namohora, that differ significantly in soil fertility. However, although fertilizer treatments were standardized by nitrogen content, they delivered substantially different amounts of P₂O₅ and K₂O. Therefore, treatment effects cannot be interpreted as reflecting fertilizer efficiency on an equivalent nutrient basis, and the stronger performance of some treatments may partly reflect differences in P and K supply rather than nitrogen-standardized fertilizer type alone.

### 4.1. Soil physicochemical responses and nutrient context

Post-harvest soil analyses revealed clear treatment-dependent nutrient accumulation patterns at both sites, reflecting differences between nutrient inputs and crop removal. Residual soil N was generally higher under CFF, 2 × CFF, and CFF + CM treatments, whereas residual soil P was highest under NPK (115.22 and 121.22 mg/kg at Tsaratanàna and Namohora, respectively). Residual soil K was highest under 2 × CFF (191 and 1290 mg/kg, respectively), indicating greater K retention following high CFF application. Higher residual Ca and Mg concentrations under CM, CFF, and CFF + CM treatments also suggest partial retention of these nutrients after harvest, while elevated Na under NPK and 2 × CFF indicates accumulation of accompanying soluble salts. Organic matter declined after cultivation at both sites but remained comparatively higher under 2 × CFF and CFF + CM, indicating greater residual organic inputs and partial organic matter retention. Overall, post-harvest soil nutrient profiles closely reflected fertilizer composition and application rates. Because the experiment was standardized on an N-equivalent basis rather than on fully balanced NPK inputs, treatment comparisons should be interpreted as the performance of the fertilizers as applied. This point is especially important for zucchini, a crop that responds strongly to phosphorus and potassium availability [37,38].

### 4.2. Agronomic responses across contrasting sites

Plant survival, plant height, leaf number, and yield all improved markedly in fertilized plots compared with the control, confirming the strong effect of nutrient addition in both agroecological contexts. However, treatment performance differed according to soil fertility status. In Tsaratanàna, the fertile coastal site, NPK produced the highest yield, although 2 × CFF and CFF + CM performed similarly. The strong response to NPK likely reflects the rapid availability of mineral nutrients, particularly phosphorus (3.27 g P/plant), under relatively favorable soil conditions. In contrast, in nutrient-poor Namohora, 2 × CFF produced the highest yield and survival, suggesting that CFF is particularly effective under low-fertility conditions, consistent with previous studies [10,11,39]. The superior performance of CFF in Namohora may be explained by both nutrient supply and improvements in soil functioning, as the 2 × CFF treatment provided higher nutrient inputs, particularly nitrogen (7.5 g/plant) and secondary nutrients such as Ca and Mg. Unlike mineral fertilizer, CFF contributes organic matter that can enhance water retention, nutrient buffering, and microbial activity, while also providing gradually mineralizable nutrients [40,41]. Insect frass may also stimulate beneficial soil microorganisms, whose increased activity can improve soil quality, nutrient availability, and plant nutrient uptake [42–45]. These effects may help reduce nutrient losses and support crop performance in poor soils. The higher contents of secondary nutrients treatment, such as Ca and Mg, from 2 × CFF may also have contributed to improved crop performance. Magnesium may have enhanced fertilizer N productivity through improved nutrient balance within the plant, while calcium likely supported root development, nutrient uptake, and stress tolerance [46]. Among the organic treatments, baseline CFF generally outperformed cattle manure for vegetative growth and yield, possibly because of faster nutrient mineralization and greater nutrient availability [40,41]. Growth and yield also increased with increasing CFF dose, consistent with findings reported in other frass studies [12,18,42,47,48]. However, this response should not be interpreted as evidence that CFF universally surpasses NPK. Rather, it indicates that higher CFF application can improve agronomic performance in this system, particularly where soil fertility is low.

### 4.3. Study limitations and future perspectives

This study has several limitations. First, the fertilizers were compared on an N-equivalent basis, but not on a fully nutrient-balanced basis; therefore, unequal inputs of phosphorus, potassium, and secondary nutrients may have contributed to treatment differences. Second, post-harvest soil data were descriptive only and were not subjected to formal statistical comparison. Third, plant tissue analysis was not performed, so nutrient uptake could not be linked directly to fertilizer inputs. Leaf area index was also not measured, so vegetative responses were assessed using plant height and leaf number only. Fourth, the experiment was conducted with three replicate blocks per site and treatment, which is limited for a field study under spatially heterogeneous conditions; although a randomized complete block design, uniform plot preparation, and border rows were used to reduce spatial variability, some residual heterogeneity may still have influenced treatment differences. Finally, the study was conducted over a single growing season, and the economic cost and local availability of insect frass fertilizer remain uncertain because farming of insects is still at an early stage. Future research should test CFF across multiple seasons and crops, include plant tissue measurements and economic analysis, and compare fertilizer treatments under more balanced nutrient inputs.

## 5. Conclusion

This study shows that cricket frass fertilizer (CFF) can improve zucchini survival, growth, and yield under field conditions in southeastern Madagascar, but its performance was site-dependent. In the nutrient-poor inland site, the 2 × CFF treatment produced the strongest overall agronomic response and the highest yield. In the more fertile coastal site, however, NPK remained the highest-yielding treatment, although 2 × CFF and CFF + CM performed similarly. These results indicate that CFF is a promising organic fertilizer, particularly under nutrient-poor conditions, but it should not be presented as a universal replacement for NPK across sites. Further multi-season studies incorporating plant tissue analysis, economic assessment, and nutrient-balanced fertilizer comparisons are needed to define its agronomic and practical value more fully.

## Supporting information

S1 Table Coefficients of variation (%) for the main response variables by site and treatment. Coefficients of variation (CV, %) were calculated as (SD / Mean) × 100 for each treatment and site combination. CV values below 30% are considered acceptable, values between 30 and 50% indicate moderate variability, and values above 50% indicate high variability. Plant survival was excluded from this assessment as it is a binary variable whose CV is inherently linked to the observed survival rate rather than to spatial variation among plots. — indicates no fruit production recorded in control plots. CV values are rounded to one decimal place.
(DOCX)
