# Supplementary material for: Assessment of cricket frass as a sustainable organic fertilizer: Effects on seedling establishment, growth and fruit yield of zucchini (Cucurbita pepo)
Source: PLoS One. 2026 Jul 6;21(7):e0351645. doi: 10.1371/journal.pone.0351645 (PMC13336191; doi:10.1371/journal.pone.0351645)
Supplement: S1 Table — Coefficients of variation (CV, %) were calculated as (SD / Mean) × 100 for each treatment and site combination. CV values below 30% are considered acceptable, values between 30 and 50% indicate moderate variability, and values above 50% indicate high variability. Plant survival was excluded from this assessment as it is a binary variable whose CV is inherently linked to the observed survival rate rather than to spatial variation among plots. — indicates no fruit production recorded in control plots. CV values are rounded to one decimal place. (DOCX) [file pone.0351645.s001.docx]

**Supporting information**

**S1 Table. Coefficients of variation (%) for the main response variables by site and treatment.**

| **Sites** | **Treatment** | **Plant height** | **Leaf number** | **Fruit length** | **Fruit diameter** | **Yield** |
| --- | --- | --- | --- | --- | --- | --- |
| Tsaratanàna | 1/4 × CFF | 25.5 | 27 | 19.3 | 20.4 | 44.9 |
| Tsaratanàna | 2 × CFF | 21.6 | 25.7 | 32 | 34.7 | 90.4 |
| Tsaratanàna | CFF | 27.1 | 23 | 26.5 | 27.3 | 73.6 |
| Tsaratanàna | CFF + CM | 36.6 | 28.5 | 37.1 | 36.5 | 101.5 |
| Tsaratanàna | CM | 27.7 | 27.2 | 27.6 | 29.4 | 79.1 |
| Tsaratanàna | Control | 25.9 | 27.1 | — | — | — |
| Tsaratanàna | NPK | 37.3 | 35.7 | 28.3 | 32.6 | 82.4 |
| Namohora | 1/4 × CFF | 36.4 | 45.7 | 22.3 | 22.3 | 46.3 |
| Namohora | 2 × CFF | 20.2 | 28 | 32.5 | 27.1 | 84.3 |
| Namohora | CFF | 23 | 26.3 | 22.1 | 21.4 | 65 |
| Namohora | CFF + CM | 31.6 | 28.3 | 31.3 | 31.4 | 88.2 |
| Namohora | CM | 30.4 | 26.2 | 18.2 | 14.6 | 46.7 |
| Namohora | Control | 39 | 32.3 | — | — | — |
| Namohora | NPK | 31.2 | 20.6 | 27.9 | 30 | 85.4 |
